# An Alternative Class of Targets for microRNAs Containing CG Dinucleotide

**DOI:** 10.3390/biology11030478

**Published:** 2022-03-21

**Authors:** Wennan Dai, Xin Su, Bin Zhang, Kejing Wu, Pengshan Zhao, Zheng Yan

**Affiliations:** 1 State Key Laboratory of Grassland Agro-Ecosystems, and College of Ecology, Lanzhou University, Lanzhou 730000, China; daiwn20@lzu.edu.cn (W.D.); sux2021@lzu.edu.cn (X.S.); 2 Computational Bioscience Research Center (CBRC), Computer, Electrical and Mathematical Sciences and Engineering Division, King Abdullah University of Science and Technology (KAUST), Thuwal 23955-6900, Saudi Arabia; bin.zhang@kaust.edu.sa; 3 Key Laboratory of Molecular Virology and Immunology, Institute Pasteur of Shanghai, Chinese Academy of Sciences, Shanghai 200031, China; kjwu@ips.ac.cn; 4 Key Laboratory of Stress Physiology and Ecology in Cold and Arid Regions, Northwest Institute of Eco-Environment and Resources, Chinese Academy of Sciences, Lanzhou 730000, China

**Keywords:** CG dinucleotide, miRNA, bulge, seed, non-canonical target

## Abstract

**Simple Summary:**

MicroRNAs are ~23 nt, highly conserved non-coding RNA molecules involved in the regulation of target gene expression. Most of the microRNA-target prediction algorithms rely heavily on seed rules and evolutionary conservation. However, such strategies suffer from missing the non-canonical target sites. The aim of this study is to identify the general features of non-canonical targets and their interactions with microRNAs. We found that the bulge-targets were preferentially associated with the microRNAs containing CG dinucleotides in their seed region. This finding indicates that non-canonical targets could be rich due to high mutation frequency of CG within the target mRNAs. Multi-step validation, which included evolutionary, overexpression, correlation, and CLASH data analysis, supports the interactome between the microRNAs with CG dinucleotides in the seed region and their bugle targets. Thus, a major novelty of this work is the identification of a sequence motif, CG dinucleotides, in the seed region of microRNAs, is strongly correlated to bulge targeting patterns.

**Abstract:**

MicroRNAs (miRNAs) are endogenous ~23 nt RNAs which regulate message RNA (mRNA) targets mainly through perfect pairing with their seed region (positions 2–7). Several instances of UTR sequence with an additional nucleotide that might form a bulge within the pairing region, can also be recognized by miRNA as their target (bugle-target). But the prevalence of such imperfect base pairings in human and their roles in the evolution are incompletely understood. We found that human miRNAs with the CG dinucleotides (CG dimer) in their seed region have a significant low mutation rate than their putative binding sites in mRNA targets. Interspecific comparation shows that these miRNAs had very few conservative targets with the perfect seed-pairing, while potentially having a subclass of bulge-targets. Compared with the canonical target (perfect seed-pairing), these bulge-targets had a lower negative correlation with the miRNA expression, and either were down-regulated in the miRNA overexpression experiment or up-regulated in the miRNA knock-down experiment. Our results show that the bulge-targets are widespread in the miRNAs with CG dinucleotide within their seed regions, which could in part explain the rare conserved targets of these miRNAs based on seed rule. Incorporating these bulge-targets, together with conservation information, could more accurately predict the entire targets of these miRNAs.

## 1. Introduction

MicroRNAs (miRNAs) are ~23 nucleotide RNAs that regulate eukaryotic gene expression post-transcriptionally [1]. MiRNAs use base-pairing to guide RNA-induced silencing complexes (RISCs) to specific message RNAs with fully or partly complementary sequences, primarily in the 3′ untranslated region [2]. The best characterized features determining animal miRNA-target recognition are six-nucleotide (nt) long seed sites, which perfectly complement the 5′ end of the miRNA (positions 2–7) [3]. This Watson-Crick seed pairing rule is sufficient on its own for predicting conserved targets above the noise of false-positive predictions in most miRNAs [4].

Most of the miRNA-target prediction algorithms rely heavily on seed rules and evolutionary conservation [5,6]. However, such strategies suffer from missing the non-canonical target sites [7]. Several biological studies have functionally validated the existence of imperfect binding sites [8,9,10]. The Ago HITS-CLIP was used to precisely map the miRNA-binding sites in both Caenorhabditis elegans [11] and mouse brains [7]. Among which, approximately one-quarter of the total binding sites did not follow the classical seed rules in mouse brains [7]. Further analysis revealed that the miR-124, one of the most abundant miRNAs in Ago complex in mouse brains, has plenty of non-canonical bulge sites. Recently, an improved CLIP-seq method, CLASH (cross linking, ligation and sequencing of hybrids), revealed around 60% of the seed interactions are non-canonical, containing bulged or mismatched nucleotides [12].

These studies strongly suggest the presence of non-canonical miRNA binding sites, but the general features of their interactions with miRNAs are largely unknown, partly due to the difficulty in determining how frequently such atypical sites are used in vivo and which kind of miRNAs might have high frequency of non-canonical binding.

Here, we analyzed a group of highly conserved miRNAs in vertebrate, but with relatively fewer conservative targets using the seed rule. We found that these miRNAs share a common feature that their seed region contains cytosine-guanine dinucleotides (hereafter refer as CG dimer). Cytosines at CG dimer are the intensive target of DNA methyltransferases in mammalian genome [13]. Spontaneous deamination of methylcytosine to thymine makes methylated cytosines unusually susceptible to mutation and/or consequent depletion [14,15]. These suggested that the lack of conserved targets for these miRNAs could be due to high evolving rate of CG-dimer within the mRNA targets. How these miRNAs and their targets cope with the CG dimer prone to mutation in the evolution? By preforming intraspecific/interspecific comparison, as well as functional and CLASH dataset analyses, we found that these miRNAs might have a subclass of targets with a bulge at the binding site compared with a fully seed-pairing. We showed that such non-canonical bulge-targets can be recognized and regulated by miRNAs. Taken together, our study uncovered a group of non-canonical miRNA targets, which have a bugle in pairing with the seed of miRNA, is widespread for miRNAs with CG dimer in the seed. This finding could increase the power for miRNA target prediction and expand our insight on the miRNA-target interaction.

## 2. Materials and Methods

### 2.1. MiRNA Sequences and 3′UTR Sequence Alignments

Mature miRNA sequences were obtained from the miRBase website (http://www.mirbase.org accessed on 9 October 2021) [16]. The broadly conserved and conserved miRNA list were obtained from TargetScanHuman (http://www.targetscan.org/cgi-bin/targetscan/data_download.vert80.cgi accessed on 9 October 2021). All miRNAs were categorized into three groups (vertebrate, mammal and primate conservative miRNA) using the miRNA Orthology Database (MirGeneDB [17] and miROrtho [18]) (Supplementary Table S1). Genomic coordinates of Ensembl human genes (hg19) were used to extract the human 3′UTR sequences and the corresponding aligned sequences from the 23-species alignment (Multiple alignment file) available at the UCSC Table browser. Only protein coding genes were included and when one gene has multiple RNA isoforms, only the one with the longest 3′UTR sequence was used in the analyses. TargetScan was used to look for the miRNAs’ canonical seed-targets based on seed rules.

### 2.2. Mutation Rate at miRNA and Target Region

We used the variants calling from human ‘1000 Genomes’ phase-3 dataset [19]. We annotated each variation using the software ANNOVAR [20], and calculated the minor allele frequencies (MAF) within the whole human population for each single nucleotide polymorphisms (SNPs). Next, The SNPs within the miRNAs and their targets were extracted. The number of mutations in CG dimer and other types of dinucleotides were calculated separately. The number of synonymous mutations at the nearby gene and the length of the gene were used to normalize the mutations in each MAF range.

### 2.3. Predictions of Seed and Bulge Target for Conservative CG Dimer miRNAs

The seed sequences for the CG dimer miRNAs were extracted to find three types of targets. Any coding gene’s 3′UTR containing a perfect complementary sequence was defined as a seed target. For the bulge-target, we allowed one extra nucleotide to exist between cytosine and thymine of a CG dimer. Randomly inserted single nucleotide in the seed sequences were used as control. The occurrences of the homologous target sites in different species were summed up for seed, bulge, and control separately as the target conservation rates.

### 2.4. MiRNAs and Target Expression Correlation Analysis

Twelve human brain prefrontal cortex samples’ miRNA (GSE29356) and coding gene transcriptome datasets (GSE22570) were used to check expression correlation. The Spearman method was used to calculate the correlation. The canonical seed-target and bulge-targets were compared with the randomly selected mRNA. The same number of genes was randomly selected 1000 times as the control group. To further validate the regulation roles of CG dimer miRNAs and bulge-targets, the in vitro miRNA overexpression datasets, including miR-126 in LM2 breast cancer cell (GSE23905), miR-184 in SY5Y (GSE26545), and miR-210 in MCF-7 cells and MDA-MB-231.cells (GSE25162), and the miRNA knock-down experiments, like miR-126 in MDA-MB-231 cells and miR-1204 in SUM159PT (GSE37185) were downloaded from GEO. The Mann-Whitney-Wilcoxon test was performed to test the seed- and bulge-targets expression change in the transfection experiments.

### 2.5. Confirm Bulge Target with Minimum Free Energy and CLASH Dataset

The RNAHybrid [21] was used to predict the minimum free energy for the miRNA-target duplex. The canonical seed-target and the bulge-target for each miRNA were compared with the randomly chosen mRNA as control.

For the CLASH dataset, the miRNA-mRNA interaction sequences were downloaded from the journal’s website in the supplementary data section [12]. In this published raw dataset, the crosslinked RNA-induced silencing complex (RISC) in HEK293 cells were immunoprecipitated. The miRNA and cognate mRNA target transcripts were ligated and sequenced together. The chimeric reads containing vertebrate conservative CG dimer miRNAs were extracted. The bulge-target was recognized if there was one extra nucleotide between the CG dimer. The original chimeric reads of miRNA and mRNA targets pairs were shuffled randomly. The scramble data were prepared by randomizing the nucleotide sequence of each chimeric reads, then remapped to the human hg19 genome. Both shuffled and scrambled data were used as random controls.

### 2.6. MiRNA and Targets Function Enrichment

We imported the broadly conserved miRNA and their canonical seed targets into the miEAA website (https://ccb-compute2.cs.uni-saarland.de/mieaa2/ accessed on 3 December 2021) [22] for functional enrichment analysis. For the bulge targets, we checked the functional enrichment for bulge targets of the vertebrate conservative miRNAs using the Metascape tool (https://metascape.org accessed on 7 December 2021) [23].

## 3. Results

### 3.1. MiRNA Containing CG Dimer Has Fewer Cononical Seed-Targets

**Figure 1:**
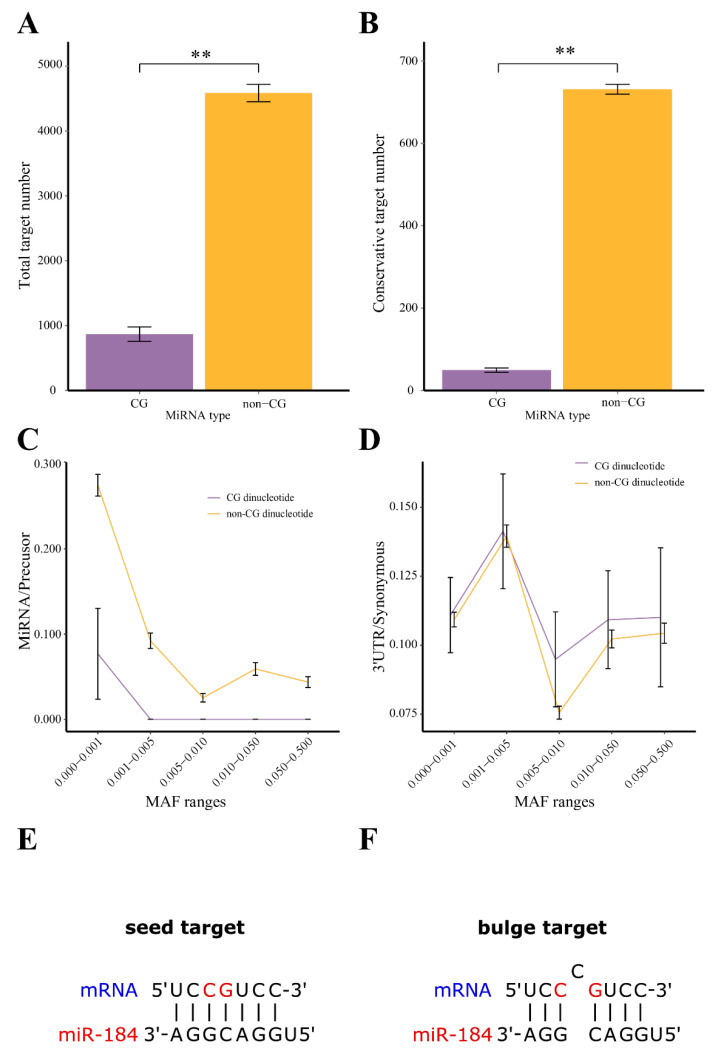
The CG dimer miRNAs have fewer conservative canonical targets than the miRNAs without CG dimer. (**A**) The number of target genes found by TargetScan for miRNAs with CG dimer at seed region or miRNAs without CG dimer. ** *p* < 0.001. (**B**) The number of conservative target genes found by TargetScan for miRNAs with CG dimer or miRNAs without CG dimer. ** *p* < 0.001. (**C**) The mutation rate of CG dimer in miRNA. The x axis is the MAF range and the y axis is the normalized number of mutations. Accumulation of miRNA mutations were normalized by the number of synonymous mutations and the length of the nearby gene. The CG dimer miRNAs group (purple line) and non-CG miRNAs group (orange line). The y axis scale is multiplied by 1000. (**D**) The mutation rate of CG dimer in the canonical seed-targets’ binding sites. Accumulation of mutation at the binding sites within 3′UTR were normalized by synonymous mutations and the mean of the total target genes’ length in different MAF ranges (x axis) for the CG dimer group (purple line) and non-CG dimer group (orange line). The y axis scale is multiplied by 1000. (**E**) The canonical seed-target model of miRNA. The miR-184 seed sequence is used to illustrate a canonical seed-target match. The mRNA is highlighted in blue, the miRNA and CG dimer are highlighted in red. (**F**) The non-canonical bulge-target model of miRNA. The miR-184 seed sequence is used to illustrate a non-canonical target match with a bulge nucleotide between the CG dimer. The mRNA is highlighted in blue, the miRNA and CG dimer are highlighted in red.

For all the human miRNA (n = 1917) annotated by miRBase [16], there are 346 miRNAs containing CG dimer at the seed region. On average, each CG miRNA has ~900 canonical targets while each non-CG containing miRNA has ~4500 targets (Figure 1A). Evolutionary conservation has been widely used to identify miRNA-binding sites together with the seed rule. The CG miRNAs have drastically fewer conservative target sites than the rest of the miRNAs (Wilcox test, *p* < 0.01, Figure 1B).

The newly derived miRNAs in human could have a lower number of targets and conservative targets. So, we used the vertebrate conserved miRNAs defined by the miRNA Orthology Database (MirGeneDB [17] and miROrtho [18]). According to the target site conservative value (Table 1), these conserved miRNAs containing CG dimer in their seed region also have much fewer conservative target sites (mean = 28) than the rest of the miRNAs (mean = 282, Wilcox test, *p* < 0.01). For the mammal and primate conservative miRNAs defined by the Orthology Database, the same class of miRNAs with CG dimer in their seed region also have much fewer conservative target sites (Supplementary Tables S2 and S3) (Wilcox test, *p* < 0.01). All these results demonstrated that CG dimer miRNAs have very few targets, and even fewer conserved targets based on the seed rule in comparison with the other miRNAs.

### 3.2. High Mutation Rate of CG Dimer at the miRNA Binding Site but Not at the miRNA Seed Region

To understand why the miRNAs with CG dimer have fewer conservative targets, we used the human ‘1000 Genomes’ project phase-3 data [19] to assess the mutation rate within miRNA and their canonical seed targets. The SNPs located within the miRNAs and their targets were extracted and the MAF of each SNPs were retrieved. We first compared the mutations for CG dimer to the non-CG dimers. The CG dimers have a low number of mutations at the low frequency range (MAF <0.001) and have no mutations at all for the rest of MAF range (Figure 1C). However, non-CG dinucleotides have a significant higher mutation than CG dimer in all frequency ranges (KS-test, *p* < 0.01, Figure 1C). The CG dimer at the targets UTR region, however, has a slightly higher mutation rates comparing with the non-CG dinucleotides (KS-test, *p* < 0.05, Figure 1D).

To compare the mutation rates between different genomic regions, we normalized the numbers of cytosines (C) converted to thymines (T) by the nearby genes’ neutral mutation number (synonymous mutation and gene length). The CG dimer within the miRNAs (<0.03) has significant lower number of C to T than the CG dimers of the binding sites at the target 3′UTR region (>1.25, KS-test, *p* < 0.01) (Figure 1C,D). In general, the CG dimers in mRNA targets have a high mutation rate, as opposed to the CG dimers in the miRNAs seed region. These results suggested that CG dimer miRNA have much fewer targets and conserved targets could be due to the high mutation rate of CG dimer within the mRNA targets.

### 3.3. Identification of Bulge Sites That Pair to miRNA Containing CG Dimer

**Figure 2:**
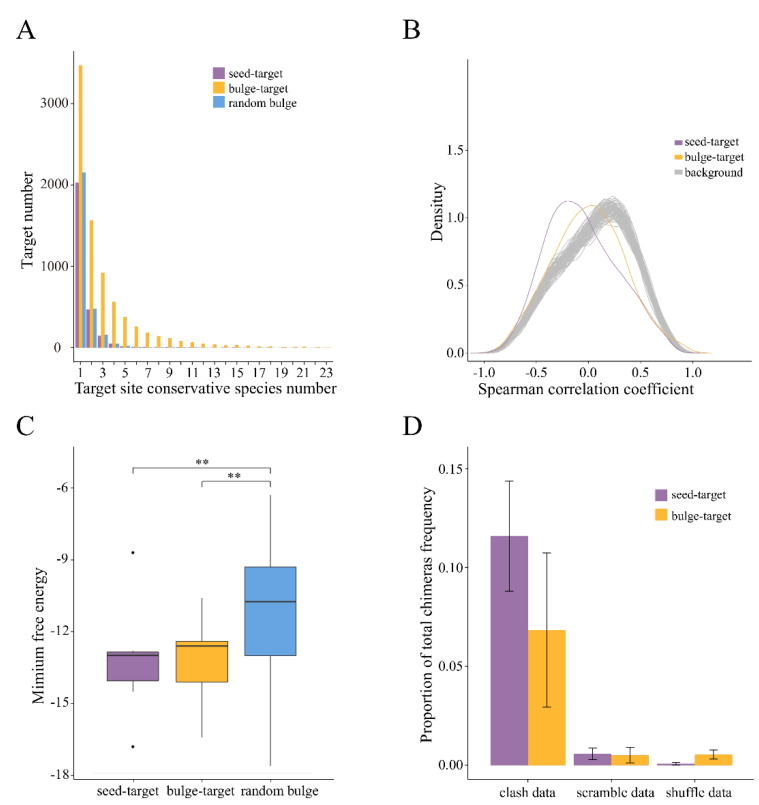
The CG dimer miRNAs and bulge-targets’ interactome. (**A**) Targets with bulge between CG dimer have higher conservative rate. The seed-targets and bulge-targets between CG dimer were compared to the random bulge control using conservation rates in 23 vertebrate species. The x axis is the conservation rates. The y axis is the number of targets for each conservation rates. (**B**) The CG dimer miRNA suppression of the bulge-targets expression. Cumulative distribution of correlation coefficient between CG dimer miRNAs and their targets expression level. (**C**) Minimum free energy between CG dimer miRNAs and seed-targets, bulge-targets, and random bulge targets. A boxplot was used to indicate the distribution of the minimum free energy for each interaction type. ** *p* < 0.001, compared with random bulge. (**D**) CLASH data validation of CG dimer miRNAs and bulge-targets interaction. The proportion of canonical seed interaction and non-canonical bulge interaction among real CLASH chimeras scramble and shuffle data.

Based on the above results, we speculate that for CG dimer miRNA, beyond the targets predicted by the seed rule, there might be an additional group of targets that don’t have binding site with perfect paring with the seed. To test this, we allow one nucleotide insertion in every position in the seed region (Figure 1E,F) for all the vertebrate conserved miRNAs with CG dimer. Using these artificial seed sequences, we find that only the bulge site inserted between the CG dimer can increase the target number and conservation of the target sites (Figure 2A). In contrast, the random bulge at the target binding site did not increase the target number nor the conservation rate compared with the canonical seed-targets (Figure 2A).

### 3.4. Transcriptome-Wide Evidence for miRNA Repression through Bulge-Target Site

To further validate these bugle-targets, we used a dataset with matched mRNA and miRNA expression values across different human ages (human age series data) [24] to quantify the correlation between CG dimer miRNAs and their bulge target at transcript level. The bulge-targets are significantly more negatively correlated to their miRNAs’ expression than the background (Wilcox test, *p* < 0.01, Figure 2B), while slightly weaker than the seed-targets, indicating that the perfect seed-pairing remains have a stronger interaction (Figure 2B).

We also used public data on transcriptome changes after over-expression or knock-down individual CG dimer miRNAs from GEO. For miR-126, miR-210, and miR-184, all the bulge-targets were significantly down-regulated after overexpression (Table 2), and in the case of the knock-down experiment for miR-1204, the bulge-targets were also much more highly expressed compared with the control genes (Wilcox test, *p* < 0.01, Table 2).

### 3.5. Minimum Free Energies of CG Bulge-Target Duplexes Are Significantly Lower Than the Random Bulges

We also compared the minimum free energy (MFE) between the canonical seed-targets, bulge-targets, and targets with random bulges using RNAhybrid [25]. The non-canonical bulge-targets have a significantly lower MFE compared with the targets with random bulge (Wilcox test, *p* < 0.05, Figure 2C). The subtle difference of MEF between seed-targets and bulge-targets indicates the bulge-targets form a strong duplex with miRNAs as the perfect seed-pairing (Figure 2C).

### 3.6. Validation of the Bulge Target Site by the CLASH Data

To allow direct mapping of miRNA-target interactions, we use the CLASH dataset [12] to validate our bulge-targets for the miRNAs containing CG dimer. Briefly, the RNA molecules present in AGO-associated miRNA-target duplexes were partially hydrolyzed, ligated, reverse transcribed, and subjected to illumina sequencing. Compared with the HITS-CLIP and PAR-CLIP dataset, CLASH technology generated a group of reads which contain the miRNAs and their target site sequence together (chimeric reads). In all the six independent CLASH experiments, we found 10 CG dimer miRNAs were detected in all the chimeric reads and eight miRNAs had, in total, 264 chimeric reads containing a bulge nucleotide between the CG dimer at the target site (Supplementary Table S4). For all miRNAs detected in the CLASH dataset, the non-canonical interactions (G.U pairs, all possible one nucleotide mismatch or bulge; non-canonical seed) were about 1.7-fold more than the perfect seed-targets. However, within the CG miRNA, only the bulge-targets between CG dimer, in comparison to randomized sequences, showed strong enrichment among all the interactions (Figure 2D). The chimeric reads strongly support the interaction between CG dimer miRNAs and bulge-targets.

## 4. Discussion

The aim of this study is to identify the general features of non-canonical miRNAs targets and their interactions with miRNA in the evolution. First, we found that the CG dimer miRNAs have surprisingly few conservative seed-targets, and these miRNAs that have been conserved over long periods of vertebrate evolution also have fewer new targets (unconservative target sites) than the other miRNAs. Therefore, we did not find evidence supporting the turnover of target sites. Selection in favor of new target sites appears to be rare: a former study found a strong signal of purifying selection against turnover of target sites [26], so the CG dimer miRNAs might exist as an alternative class of targets. Secondly, we found a significant higher CG mutation frequency at the miRNAs binding sites but not at the miRNA itself. This result suggests the mutations at the CG dimer miRNA target sites may be relatively frequent. It follows that the mutation at the binding sites might be neutral in some of these cases. This reinforces our initial hypothesis that these CG dimer miRNAs have bulge-nucleotides to tolerate target binding site mutation.

The chimeric reads from CLASH data strongly support the interaction between CG dimer miRNAs and bulge-targets. Compared with the seed-targets, however, these bulge-targets had a weaker negative correlation with the miRNA expression, indicating that the perfect seed-pairing remains have stronger interaction. This is consistent with structural accessibility of target sequences, total free energy of miRNA-target hybridization, and topology of base-pairing.

The lack of canonical seed-targets makes functional enrichment impossible for each CG dimer miRNA. This makes hard to predict the CG dimer miRNA function. Even pooling all the CG dimer miRNAs together, we only got fewer than one hundred seed-targets. The bulge-targets, however, were much more abundant than the seed-targets. So, we could successfully perform GO analysis for single CG dimer miRNAs. For instance, the bulge-targets for three CG dimer miRNAs (miR-126, miR-184, and miR-187) are functionally enriched in the synapse/neuron projection and the RHO GTPase cycle, indicating that fast evolved neuron cells [27,28] and GTP-GDP cycling [29,30] are most sensitive to the perturbation of seed complementary base-pairing and most receptive in accommodating evolutionary innovation, such as bulge-target recognition. Thus, a major novelty of this work is not only that the identification of a sequence motif, CG dimer, in the seed region of miRNAs, is strongly correlated to bulge targeting patterns, but also the improvement of functional enrichment for the targets for individual miRNAs.

## 5. Conclusions

Overall, we found that the bulge-targets were preferentially associated with the miRNAs containing CG dimer in their seed region. Multi-step validation, which included evolutionary, overexpression, correlation, and CLASH data analysis supports the possibility that within the miRNAs with CG dimer in the seed region, there is a group of targets containing a bugle in the binding site.

## Figures and Tables

**Table 1:** Vertebrate conservative miRNA TargetScan result.

Seed Sequence	MicroRNA	Conservative Target Number	Percent	Total Target
*CG*UUUGC	hsa-mir-1282	1	0.0025	398
*CG*UGUCU	hsa-mir-187	3	0.0068	439
C*CG*GUUC	hsa-mir-671-3p	5	0.0175	285
AAC*CG*UU	hsa-mir-451	14	0.0433	323
*CG*UAC*CG*	hsa-mir-126	17	0.1328	128
GGA*CG*GA	hsa-mir-184	21	0.0363	578
AA*CG*GAA	hsa-mir-191	32	0.0691	463
CUCUAGC	hsa-mir-1251	57	0.0348	1636
AUACCUC	hsa-mir-875-5p	64	0.0530	1207
GAGUUGA	hsa-mir-219-1-3p	65	0.0426	1525
CAUGGAU	hsa-mir-490-5p	75	0.0402	1866
GGGUCUU	hsa-mir-193a-5p	78	0.0404	1933
GAAUUGU	hsa-mir-219-2-3p	93	0.0546	1704
GAUCAGA	hsa-mir-383	101	0.0645	1565
AACCUGG	hsa-mir-490-3p	102	0.0519	1964
AUCACUA	hsa-mir-34c-3p	111	0.0645	1720
AUGACAC	hsa-mir-425	112	0.0566	1978
GGAGUGU	hsa-mir-122	116	0.0463	2503
AUGUGCC	hsa-mir-455-5p	128	0.0790	1620
UUGUU*CG*	hsa-mir-375	136	0.0756	1799
UUGUGUC	hsa-mir-599	138	0.0588	2347
UGACAUC	hsa-mir-489	138	0.0668	2067
AGUAGUU	hsa-mir-1244	140	0.0987	1419
CAUAUUG	hsa-mir-1279	146	0.0636	2296
AAUCUCA	hsa-mir-216a	153	0.0681	2248
CAGGAAC	hsa-mir-873	167	0.0524	3184
AAUGCCC	hsa-mir-365	171	0.1050	1628
AAUCUCU	hsa-mir-216b	172	0.0814	2113
CAGUCCA	hsa-mir-455-3p	181	0.0816	2217
CCAGCAU	hsa-mir-338-3p	189	0.0691	2736
GUCAGUU	hsa-mir-223	193	0.1051	1837
GGAAGCC	hsa-mir-671-5p	201	0.0806	2493
AAUCACU	hsa-mir-34b	217	0.0939	2312
ACUGCAU	hsa-mir-217	223	0.0985	2265
ACCACAG	hsa-mir-140-3p	223	0.0843	2644
GGCAAGA	hsa-mir-31	227	0.0985	2305
AGUGGUU	hsa-mir-140-5p	237	0.1424	1664
GUAGUGU	hsa-mir-142-3p	241	0.1971	1223
GAGGUAG	has-mir-98	243	0.1753	1386
GUAACAG	hsa-mir-194	246	0.1118	2200
UAAUGCU	hsa-mir-155	268	0.1419	1888
AUGGCAC	hsa-mir-183	283	0.1429	1980
CCUUCAU	hsa-mir-205	284	0.1115	2548
GGAAGAC	hsa-mir-7	286	0.1004	2848
AGCUGCC	hsa-mir-22	308	0.1381	2231
UUUUUGC	hsa-mir-129-5p	334	0.0827	4040
GCUGGUG	hsa-mir-138	368	0.1531	2403
CUGGAAA	hsa-mir-875-3p	412	0.1135	3629
GGCUCAG	hsa-mir-24	417	0.1206	3458
AAAGAAU	hsa-mir-186	498	0.1149	4333
UCCAGUU	hsa-mir-145	504	0.1980	2545
UGCAUAG	hsa-mir-153	515	0.2702	1906
AUAAAGU	hsa-mir-142-5p	516	0.1655	3117
GAGGUAU	hsa-mir-202	569	0.3506	1623
ACAGUAC	hsa-mir-101	582	0.2358	2468
ACAAUAU	hsa-mir-338-5p	587	0.1952	3007
ACAGUAU	hsa-mir-144	616	0.2290	2690
UGUGCUU	hsa-mir-218	665	0.2847	2336
UAUUGCU	hsa-mir-137	817	0.3719	2197
CUUUGGU	hsa-mir-9	893	0.3323	2687

The results were sorted from the lowest to the highest conservative rates. The CG dimers are highlighted in red letters.

**Table 2:** Functional analyses of CG dimer miRNAs in the transfection data.

MiRNA	Cell Line	Experiment	Target Type	*p* Value
miR-126	LM2 breast cancer cell	over-expression	seed	9.18 × 10^−6^
miR-126	LM2 breast cancer cell	over-expression	bulge	3.49 × 10^−9^
miR-1204	SUM159PT	knock down	seed	2.43 × 10^−2^
miR-1204	SUM159PT	knock down	bulge	5.01 × 10^−3^
miR-210	MCF7	over-expression	seed	0.8669572
miR-210	MCF7	over-expression	bulge	0.03873948
miR-210	MDA.MB.231	over-expression	seed	1.31 × 10^−41^
miR-210	MDA.MB.231	over-expression	bulge	1.36 × 10^−93^
miR-184	SY5Y	over-expression	seed	5.32 × 10^−14^
miR-184	SY5Y	over-expression	bulge	1.70 × 10^−7^

## Data Availability

Not applicable.

## Supplementary Materials

The following supporting information can be downloaded at: https://www.mdpi.com/article/10.3390/biology11030478/s1. Table S1: All the 23 species which were used for miRNA and target conservation analysis; Table S2: Mammal conservative miRNA TargetScan result; Table S3: Primate conservative miRNA TargetScan result; Table S4: CLASH chimeras reads which the miRNA-bulge-target duplex were sequenced.
